# Precision Synthesis of Functional Polysaccharide Materials by Phosphorylase-Catalyzed Enzymatic Reactions

**DOI:** 10.3390/polym8040138

**Published:** 2016-04-11

**Authors:** Jun-ichi Kadokawa

**Affiliations:** Graduate School of Science and Engineering, Kagoshima University, 1-21-40 Korimoto, Kagoshima 890-0065, Japan; kadokawa@eng.kagoshima-u.ac.jp; Tel.: +81-99-285-7743; Fax: +81-99-285-3253

**Keywords:** amylose, enzymatic reaction, phosphorylase, polysaccharide, polymerization, supramolecule

## Abstract

In this review article, the precise synthesis of functional polysaccharide materials using phosphorylase-catalyzed enzymatic reactions is presented. This particular enzymatic approach has been identified as a powerful tool in preparing well-defined polysaccharide materials. Phosphorylase is an enzyme that has been employed in the synthesis of pure amylose with a precisely controlled structure. Similarly, using a phosphorylase-catalyzed enzymatic polymerization, the chemoenzymatic synthesis of amylose-grafted heteropolysaccharides containing different main-chain polysaccharide structures (e.g., chitin/chitosan, cellulose, alginate, xanthan gum, and carboxymethyl cellulose) was achieved. Amylose-based block, star, and branched polymeric materials have also been prepared using this enzymatic polymerization. Since phosphorylase shows a loose specificity for the recognition of substrates, different sugar residues have been introduced to the non-reducing ends of maltooligosaccharides by phosphorylase-catalyzed glycosylations using analog substrates such as α-d-glucuronic acid and α-d-glucosamine 1-phosphates. By means of such reactions, an amphoteric glycogen and its corresponding hydrogel were successfully prepared. Thermostable phosphorylase was able to tolerate a greater variance in the substrate structures with respect to recognition than potato phosphorylase, and as a result, the enzymatic polymerization of α-d-glucosamine 1-phosphate to produce a chitosan stereoisomer was carried out using this enzyme catalyst, which was then subsequently converted to the chitin stereoisomer by *N*-acetylation. Amylose supramolecular inclusion complexes with polymeric guests were obtained when the phosphorylase-catalyzed enzymatic polymerization was conducted in the presence of the guest polymers. Since the structure of this polymeric system is similar to the way that a plant vine twines around a rod, this polymerization system has been named “vine-twining polymerization”. Through this approach, amylose supramolecular network materials were fabricated using designed graft copolymers. Furthermore, supramolecular inclusion polymers were formed by vine-twining polymerization using primer–guest conjugates.

## 1. Introduction

**Figure 1:**
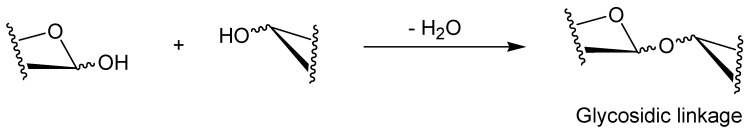
Formation of glycosidic linkage between anomeric hydroxy group of sugar residue and hydroxy group of another sugar residue.

Polysaccharides are commonly present in nature and have been identified as vital materials for important *in vivo* functions [1]. These molecules are composed of monosaccharide residues linked together through glycosidic bonds, a type of covalent linkage that joins a monosaccharide residue at its anomeric position to another group, typically another saccharide moiety, such that it is structurally a result of dehydrative condensation between the components’ two hydroxy groups (Figure 1) [2,3,4]. Natural polysaccharides are constructed by a wide variety of monosaccharide repeating units linked through the different stereo- and region-types of glycosidic bonds, giving rise to quite complicated chemical structures. The structural diversity of polysaccharides leads to the provision of a whole range of biological functions, and a subtle change in their structure has a profound effect on the properties and functions of these molecules [5]. For example, two representative natural polysaccharides, cellulose and starch, are both composed of glucose repeating units, but are linked through stereo-oppositely β(1→4)- and α(1→4)-glucosidic bonds, respectively [6,7]. Owing to the differences in the stereochemistry of the linkages between the same glucose repeating units, the roles of cellulose and starch in nature are completely different, such that, the former is a structural material and the latter acts as an energy provider as a component of starch, respectively. The precise synthesis of structurally well-defined polysaccharides composed of stereo- and regio-controlled glycosidic linkages has therefore attracted much attention in carbohydrate and materials sciences in the development of new functional bio-related materials.

To provide well-defined chain structures from monosaccharide repeating units, the stereo- and regio-selective formation of glycosidic linkages between two glycosyl donor and acceptor substrates, a so-called ”glycosylation”, is in demand [2,3,4]. In a typical glycosylation reaction, an anomeric carbon of the donor substrate is activated by the introduction of a leaving group to the molecule, such that it can react with the free hydroxy group of the acceptor substrate. It is important that the other hydroxy groups of the glycosyl donor and acceptor are protected to prevent unwanted side reactions from occurring. For the stereo- and regio-selective formation of the glycosidic linkage, the leaving group, protecting groups, catalyst, and solvent are therefore important and must be selected appropriately. The control of the stereo- and regio-selectivities of the resulting glycosidic linkages in general chemical glycosylations is still a challenging research topic in glycoscience.

**Figure 2:**
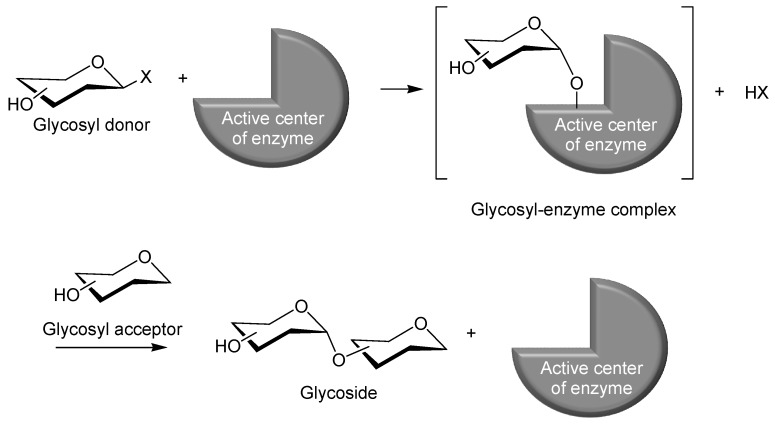
Reaction scheme for enzymatic glycosylation.

An alternative to chemical glycosylations that has also been developed involves an *in vitro* approach, the so-called “enzymatic glycosylation”, which offers advantages because enzymatic reactions are superior to chemical catalysis in terms of stereo- and regio-selectivities [8,9]. Of the six main classes of enzymes, transferase (glycosyl transferase) and hydrolase (glycosyl hydrolase) have been used successfully as catalysts in enzymatic glycosylations [10,11]. In these reactions, a glycosyl donor and a glycosyl acceptor are employed in their unprotected forms, whereby the reaction progresses in a stereo- and regio-controlled fashion by enzymatic catalysis in aqueous media [9]. In the same fashion as the aforementioned chemical glycosylation, the enzymatic formation of a glycosidic linkage occurs between the anomeric position of one monosaccharide residue, the activated glycosyl donor, and the hydroxy group of the second monosaccharide residue, the glycosyl acceptor (Figure 2). In the course of the reaction, the anomeric position of the glycosyl donor is initially recognized by and interacts with the catalytic center of the enzyme to form a glycosyl–enzyme complex. Next, the hydroxy group of the glycosyl acceptor attacks the anomeric carbon of the complex in a stereo- and regio-selective manner, according to the specificity of the enzyme, which gives rise to the direct formation of the unprotected glycoside. As mentioned previously, the enzymes that have been employed to catalyze these glycosylations are primarily glycosyl transferase and glycosyl hydrolase. Additionally, the former can be sub-classified into the synthetic enzymes (Leloir glycosyl transferase) phosphorolytic enzyme (phosphorylase), and sucrase [12,13].

Due to the fact that polysaccharides are theoretically produced by repeated glycosylations, several enzymatic approaches have been employed in the efficient syntheses of polysaccharides (enzymatic polymerizations) [14,15]. Phosphorylase is an enzyme that has been employed to synthesize amylose with a precise stereo- and regio-controlled structure [16]. This enzymatic approach provides a pure amylose sample, which is useful as the separation of amylose from amylopectin in natural starch is quite difficult. Furthermore, as a result of the loose specificity for the recognition of substrates by phosphorylase, functional non-natural polysaccharides other than amylose have also been synthesized by phosphorylase catalysis. In this review article, on the basis of the above viewpoints and background, the precise syntheses of functional polysaccharide materials by phosphorylase-catalyzed enzymatic reactions are reviewed.

## 2. Characteristic Features of Phosphorylase Catalysis

**Figure 3:**
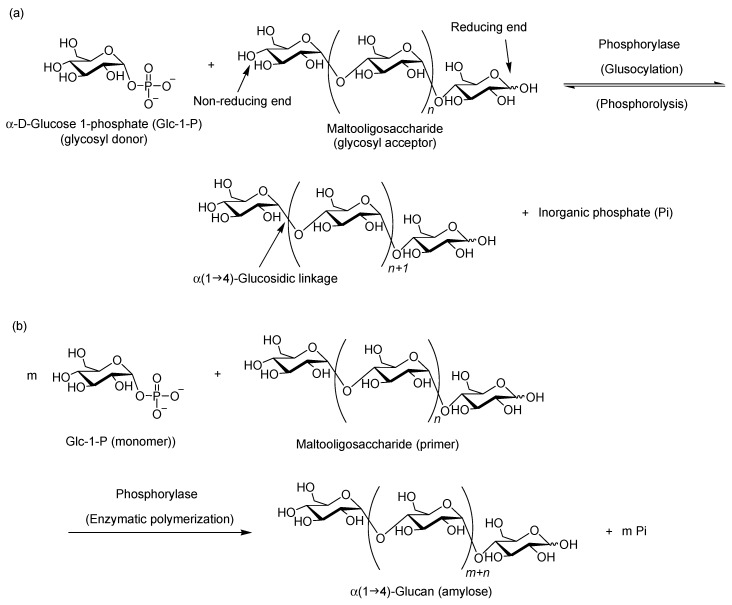
Phosphorylase-catalyzed (**a**) phosphorolysis and glucosylation and (**b**) enzymatic polymerization.

Phosphorylase, belonging to the GT35 family (EC 2.4.1.1), is often called starch phosphorylase, glycogen phosphorylase, or α-glucan phosphorylase. It is well accepted, however, that this enzyme is simply referred to as “phosphorylase”, because it is the most extensively studied and widely used enzyme as the catalyst for reactions in many of the phosphorylases found in nature. In nature, phosphorylase catalyzes the phosphorolytic cleavage of α(1→4)-glucans, such as amylose and glycogen, at their non-reducing ends in the presence of inorganic phosphate (Pi) to produce α-d-glucose 1-phoshate (Glc-1-P) (Figure 3a) [17,18,19]. Because of the reversibility of this reaction, phosphorylase also catalyzes enzymatic glucosylation reaction using Glc-1-P as the glycosyl donor (Figure 3a) [13], whereby a glucose residue is transferred from Glc-1-P to the non-reducing end of the glycosyl acceptor to form an α-(1→4)-glucosidic linkage. Maltooligosaccharides with degrees of polymerization (DPs) higher than the smallest one, which are recognized by phosphorylase, are typically used as glycosyl acceptors. The smallest substrates used in phosphorolysis and glucosylation reactions catalyzed by the most common phosphorylase isolated from potato (potato phosphorylase) are typically maltopentaose (Glc_5_) and maltotetraose (Glc_4_). On the other hand, Glc_4_ and maltotriose (Glc_3_) are the smallest substrates recognized in phosphorolysis and glucosylation reactions by the catalysis of phosphorylase isolated from thermophilic bacterial sources (thermostable phosphorylase) [20,21,22,23]. These observations suggest that the sources of phosphorylases strongly affect recognition behavior of the substrates.

When an excess molar ratio of Glc-1-P with respect to the maltooligosaccharide acceptor is present in the reaction system, the successive glucosylations in the presence of phosphorylase occur as a propagation of polymerization to yield the α(1→4)-glucan polymer, *i.e.*, amylose (Figure 3b) [20,24,25,26]. The reaction of such phosphorylase-catalyzed successive glucosylations is a chain-growth polymerization, and the glycosyl acceptor is often referred to as the “primer” of the polymerization because the reaction is initiated at the non-reducing end of the acceptor. Since the phosphorylase-catalyzed polymerization is conceived analogously to a living polymerization, the molecular weight of the generated amylose can be controlled by the monomer (Glc-1-P)/primer feed ratios, and the distribution of polymeric products is typically narrow (*M*_w_/*M*_n_ < 1.2) [27]. 

**Figure 4:**
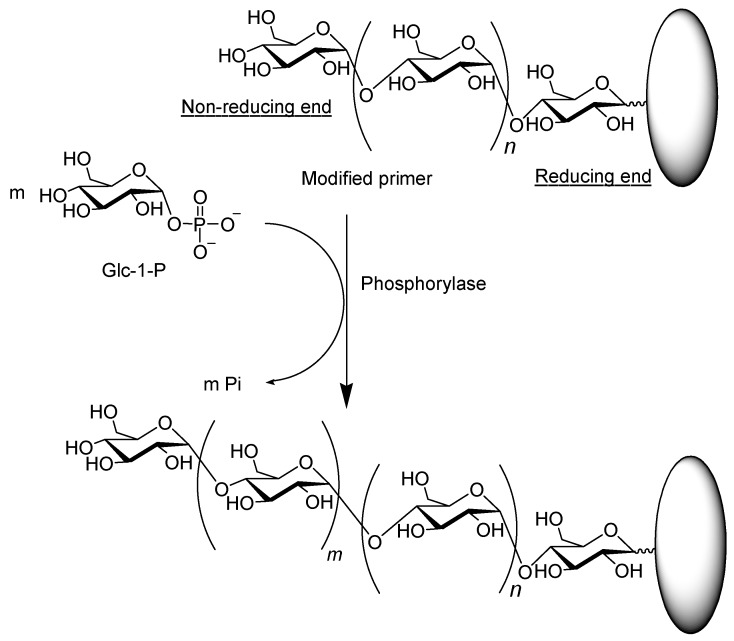
Phosphorylase-catalyzed enzymatic polymerization using modified primer.

The phosphorylase-catalyzed enzymatic polymerization can be conducted using modified maltooligosaccharide primers, which are covalently immobilized on other substances at its reducing ends, such as a polymeric chain, which does not take part in the polymerization (Figure 4) [28,29,30,31,32,33,34]. Such a modified primer is typically multifunctional because of the presence of multiple non-reducing maltooligosaccharide chain ends on the substance, and has produced various amylose-grafted polymeric materials, such as amylose-grafted polystyrene, polyacetylene, poly(dimethylsiloxane), poly(vinyl alcohol), and poly(l-glutamic acid) [35,36,37,38,39,40,41]. Amylose-grafted brushes were also obtained by the phosphorylase-catalyzed enzymatic polymerization of Glc-1-P from maltoheptaose primers covalently attached to Au and Si surfaces [42].

**Figure 5:**
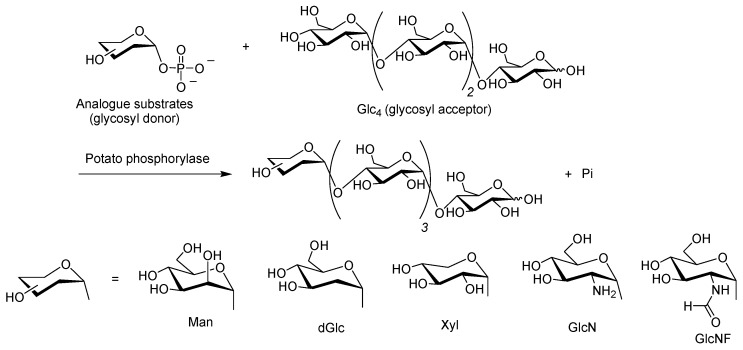
Phosphorylase-catalyzed enzymatic glycosylations using analogue substrates as glycosyl donors to produce non-natural pentasaccharides.

Phosphorylase has demonstrated loose specificity for the recognition of the substrate structures of interest, and therefore, the extension of phosphorylase-catalyzed enzymatic glucosylations using several monosaccharide 1-phosphates, *i.e.*, analog substrates of Glc-1-P, as glycosyl donors to obtain non-natural oligosaccharides comprising the corresponding monosaccharide residues at the non-reducing ends has been investigated [43,44]. For example, potato phosphorylase has shown recognition for α-d-mannose, 2-deoxy-α-d-glucose, α-d-xylose, α-d-glucosamine, and *N*-formyl-α-d-glucosamine 1-phosphates (Man-1-P, dGlc-1-P, Xyl-1-P, GlcN-1-P, and GlcNF-1-P, respectively) as glycosyl donors in glycosylations using Glc_4_ as a glycosyl acceptor, to produce non-natural α-mannosylated, 2-deoxy-α-glucosylated, α-xylosylated, α-glucosaminylated, and *N*-formyl-α-glucosaminylated pentasaccharides, respectively (Figure 5) [45,46,47,48,49,50].

## 3. Synthesis of Amylose-Containing Functional Polysaccharide Materials by Phosphorylase-Catalyzed Enzymatic Polymerization

Besides linear natural polysaccharides such as cellulose, chitin/chitosan, and amylose, branched or grafted structures, where a main-chain polysaccharide is functionalized with other polysaccharide side chains by covalent linkages [51], have also often been observed in naturally occurring polysaccharides. Such chemical structures likely contribute to promising and specific functions and demonstrate their important roles in natural processes. Efficient methods for the preparation of well-defined branched or grafted artificial polysaccharides have therefore attracted increasing attention in the glycomaterial research fields.

**Figure 6:**
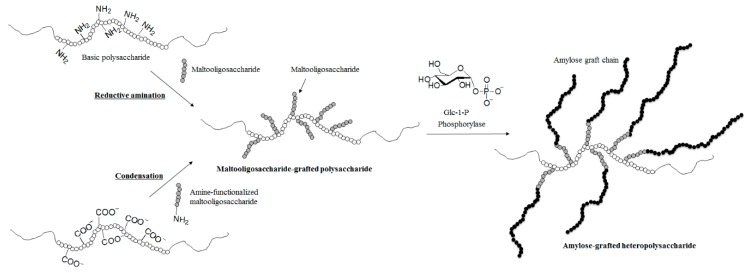
Chemoenzymatic synthesis of amylose-grafted heteropolysaccharides via reductive amination and condensation, followed by phosphorylase-catalyzed enzymatic polymerization.

On the basis of the above viewpoint, the phosphorylase-catalyzed enzymatic polymerization of Glc-1-P has been employed for the synthesis of amylose-grafted functional heteropolysaccharide materials by chemoenzymatic approaches (Figure 6) [29,30,31,32,33,34]. One such synthesis was achieved using the aforementioned modified primer, where the reducing ends of maltooligosaccharides were covalently connected to polysaccharide chains, such that, in this case, appropriate chemical reactions were carried out to immobilize maltooligosaccharides onto the main-chain polysaccharides, and the resulting modified primers were then used for the phosphorylase-catalyzed enzymatic polymerization. Two types of chemical reactions have been employed to prepare the modified primers: the reductive amination using reductants of maltooligosaccharides with basic polysaccharides containing amino groups, and the condensation using condensing agents of amine-functionalized maltooligosaccharides with acidic polysaccharides containing carboxylate groups.

Using the former approach, chitosan, a basic polysaccharide with amino groups at the C-2 position in its glucosamine repeating units, was functionalized to form a maltooligosaccharide-grafted chitosan. This material was then converted into a maltooligosaccharide-grafted chitin by *N*-acetylation, using acetic anhydride, of the amino groups in the chitosan main-chain. Finally, from the maltooligosaccharide primer ends of the chitin/chitosan derivatives, amylose chains were elongated by the phosphorylase-catalyzed enzymatic polymerization of Glc-1-P to yield amylose-grafted chitin/chitosan [52,53]. When the polymerization mixture was slowly dried at 40–50 °C, a hydrogel of the amylose-grafted chitosan was produced.

An amylose-grafted cellulose was also synthesized using a similar synthetic approach [54]. A cellulose derivative with partial conversion of its C-6 hydroxy groups to amine groups was initially prepared by the successive partial tosylation of its C-6 hydroxy groups, displacement of the tosylates by azido groups, and their subsequent reduction to amine groups. This amine-functionalized cellulose was then reacted with a maltooligosaccharide by reductive amination to give a primer maltooligosaccharide-grafted cellulose, which was further reacted with Glc-1-P in a phosphorylase-catalyzed enzymatic polymerization to produce the amylose-grafted cellulose of interest. The product formed has a very unique structure because it is made up of two representative glucose polymers, cellulose and amylose, which are composed of the same repeating units but linked through stereoregular and opposite glucosidic bonds, β(1→4)– and α(1→4)–, respectively. When the polymerization solution was kept on a Petri dish in an ambient atmosphere for several days, it was converted entirely to a hydrogel, which was stronger than the aforementioned amylose-grafted chitosan hydrogel. This drying technique produceda solid (or film) material. Moisturizing the film returned the molecule to its hydrogel form again, a process that could be reversibly switched by the subsequent drying and wetting of the system.

Alternatively, the latter condensation reaction was also used to prepare modified primers from acidic polysaccharides, such as alginate, xanthan gum, and carboxymethyl cellulose (CMC) [55,56,57,58]. The reaction of a maltooligosaccharide lactone with 2-azidoethylamine and the subsequent reduction of the azido group using NaBH_4_ gave an amine-functionalized maltooligosaccharide at the reducing end. Next, maltooligosaccharide-grafted alginate, xanthan gum, and CMC were obtained by condensation with the carboxylates of the corresponding polysaccharides using the condensing agents of water-soluble carbodiimide (WSC) and *N*-hydroxysuccinimide (NHS). Finally, the phosphorylase-catalyzed enzymatic polymerization of Glc-1-P with these maltooligosaccharide primer chains was carried out to obtain amylose-grafted alginate, xanthan gum, and CMC. The resulting amylose-grafted CMC formed a film upon the drying of a thinly spread alkaline solution (0.040 g in 0.50 mol/L aqueous NaOH (1.50 mL)). Scanning electron microscopy (SEM) imaging of the film showed a surface morphology comprised of highly entangled nanofibers. After removal of the residual NaOH from the film by immersion in water, further SEM imaging showed that the nanofibers had merged at the interface, while the fiber arrangement was retained in the bulk of the material. This method offers an efficient self-assembling generative (bottom-up) route that achieves control over the morphology and self-assembly of heteropolysaccharides to fabricate new nanofibrillated materials. 

**Figure 7:**
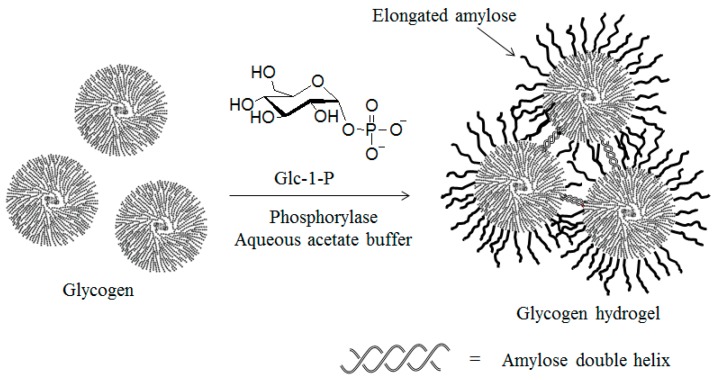
Phosphorylase-catalyzed enzymatic polymerization using glycogen as multifunctional primer to produce hydrogel.

Glycogen is known to be a water soluble and high molecular-weight natural polysaccharide, composed of linear α(1→4)-glucan chains containing an average of 10 to 14 glucose residues, which are further interlinked by α(1→6)-glucosidic linkages that lead to a highly branched structure [59,60]. Aside from glycogen’s role in *in vivo* phosphorolysis with glycogen phosphorylase, it is also used as a multifunctional primer for phosphorylase-catalyzed enzymatic polymerizations because of the presence of a number of non-reducing α(1→4)-glucan chain ends [61]. When the phosphorylase-catalyzed enzymatic polymerization of Glc-1-P with glycogen was carried out in aqueous acetate buffer solution, followed by the standing of the reaction mixture under ambient atmosphere for 24 h, the solution was fully converted into a hydrogel (Figure 7). This hydrogelation was induced by the formation of cross-links as a result of the double helix conformation of the elongated amylose chains among the glycogen molecules [62,63]. The stress-strain curves under compressive mode of the hydrogels obtained by employing different Glc-1-P/glycogen ratios showed that the gels were strengthened and then became brittle with increasing amounts of glycogen. The formation of more double helix cross-linking points from the larger number of elongated amylose chains induced the increase of the gel strength when the amounts of glycogen increased. However, the further larger number of cross-linking points most likely led to the brittle nature of the gels. The hydrogels were readily converted to cryogels by lyophilization. The stress-strain curves under compressive mode of these cryogels showed that harder cryogels were obtained with increases in the amount of glycogen used for the initial hydrogelation. This result is probably due to the formation of smaller networks when larger numbers of cross-linking points were present with larger amounts of glycogen in the system. Furthermore, a transparent film could be obtained by casting the alkaline solution of the cryogel on a glass plate, followed by drying.

Glc_5_-*block*-alkyl chain surfactants (octyl, C_8_Glc_5_; dodecyl, C_12_Glc_5_; hexadecyl, C_16_Glc_5_) formed micelles in water, which dissociated upon phosphorylase-catalyzed enzymatic polymerization. Moreover, the micelle-to-vesicle transition of the mixed lipid/primer systems was controlled by the enzymatic polymerization. An enzyme-responsive artificial chaperone system using C_12_Glc_5_ and phosphorylase b was also designed to facilitate protein refolding. After both guanidine hydrochloride and heat denaturation of carbonic anhydrase B, the efficient refolding of the protein was observed upon the controlled association of the protein molecules and the C_12_Glc_5_ micelle after enzymatic polymerization [64,65]. The poly(l-lysine) moiety with pendant maltooligosaccharide primers and cholesterols formed positively-charged nanogels via self-assembly in water, which after phosphorylase-catalyzed enzymatic polymerization with Glc-1-P gave amylose-conjugated nanogels [66]. A series of amylose-based star polymers (1, 2, 4, and 8 arms) was also synthesized by phosphorylase-catalyzed enzymatic polymerization using Glc_5_-functionalized poly(ethylene glycol) (PEG) [67]. The eight-arm primer formed was able to act as a gelator when triggered enzymatically.

## 4. Synthesis of Non-Natural Functional Polysaccharide Materials by Phosphorylase-Catalyzed Enzymatic Reactions Using Analog Substrates

**Figure 8:**
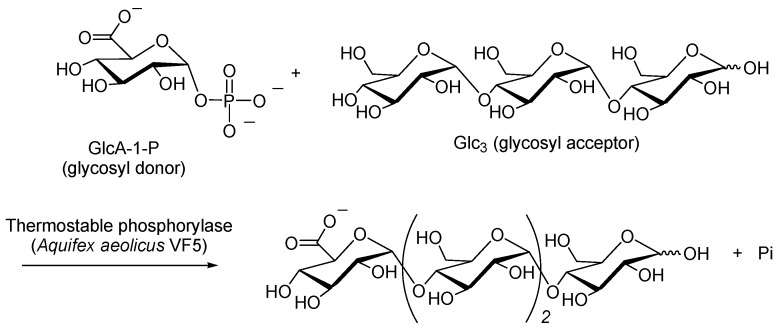
Thermostable phosphorylase-catalyzed enzymatic glucuronylation using GlcA-1-P as glycosyl donor to produce acidic tetrasaccharide.

In addition to the introduction of GlcN units containing an amine functional group to maltooligosaccharides by phosphorylase-catalyzed glycosylation (glucosaminylation) using GlcN-1-P as a glycosyl donor [49], an attempt has also been made to enzymatically produce a different functional oligosaccharide by phosphorylase catalysis using the analog substrate, α-d-glucuronic acid 1-phosphate (GlcA-1-P), in which a carboxylate group can be introduced to the maltooligosaccharide. Potato phosphorylase did not recognize GlcA-1-P, however, and thus the desired functional oligosaccharide was not produced by catalysis with this particular enzyme. Interestingly, it was found that thermostable phosphorylase isolated from *Aquifex aeolicus* VF5 [68] did recognize GlcA-1-P and could catalyze the glycosylation (glucuronylation) of Glc_3_, the smallest glycosyl acceptor recognized by this enzyme, to produce an acidic tetrasaccharide containing a carboxylate group (Figure 8) [69].

**Figure 9:**
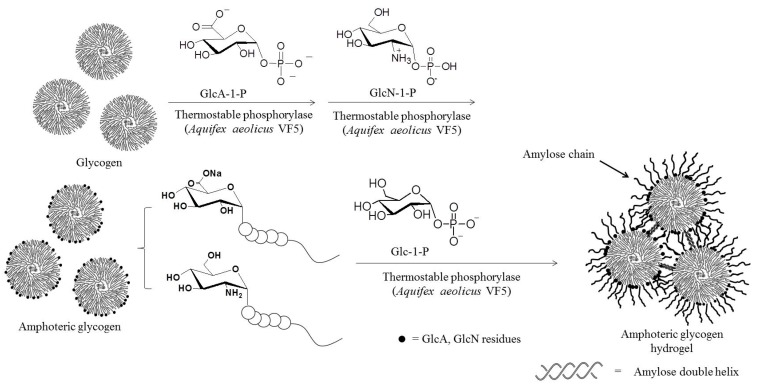
Phosphorylase-catalyzed successive enzymatic reactions to produce amphoteric glycogen hydrogel.

Through the use of the above phosphorylase-catalyzed glucosaminylation and glucuronylation reactions using analog substrates (GlcN-1-P and GlcA-1-P), highly branched amphoteric polysaccharides, such as amphoteric glycogens having both basic GlcN and acidic GlcA residues, could also be synthesized (Figure 9) [70,71]. The thermostable phosphorylase-catalyzed glucuronylation of highly branched polysaccharides using GlcA-1-P was first conducted to give rise to acidic materials [72], followed by thermostable phosphorylase-catalyzed glucosaminylation of the products using GlcN-1-P to obtain amphoteric polysaccharides. The functionalities of the GlcA/GlcN residues depended on the feed ratios employed in the reaction. The electrokinetic ζ-potential values of the amphoteric products changed from positive to negative as the pH of the solution increased. The values of the isoelectric points of the amphoteric products could be calculated by the pH values observed when the ζ-potential became zero, and not surprisingly, they were dependent on the GlcA/GlcN ratios present in the products. Amphoteric glycogen hydrogels could be prepared by the formation of cross-links between the double-helical conformations of the elongated amylose chains upon the thermostable phosphorylase-catalyzed enzymatic polymerization of Glc-1-P with the non-functionalized, non-reducing ends of the amphoteric glycogens (Figure 9) [71]. The produced hydrogels showed pH-responsive behavior, where they were soluble in alkaline conditions and returned to hydrogels upon acidification of the system. Furthermore, the hydrogels exhibited pH-dependent shrinking/swelling behavior.

**Figure 10:**
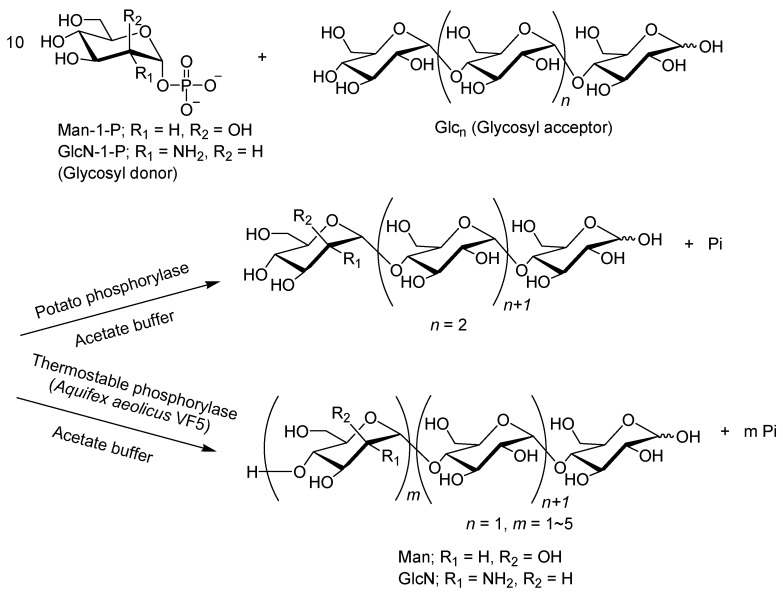
Differences in glycosylation using Man-1-P/GlcN-1-P as glycosyl donors by potato and thermostable phosphorylase catalyses.

A series of studies on the potato phosphorylase-catalyzed glycosylations using analog substrates have suggested that if a single monosaccharide residue is transferred from the analog substrates to the non-reducing end of the maltooligosaccharide acceptor, further glycosylations will not occur because the new structure different from the Glc residue at the non-reducing end is no longer recognized by the enzyme (Figure 10) [43,44]. When the thermostable phosphorylase (from *Aquifex aeolicus* VF5) was instead employed, reactions of Glc_3_ using excess molar ratios of Man-1-P and GlcN-1-P, successive mannosylations/glucosaminylations took place to obtain non-natural heterooligosaccharides composed of α(1→4)-linked mannose/glucosamine chains (Figure 10) [73]. The MALDI-TOF mass spectra of the products of this thermostable phosphorylase-catalyzed enzymatic glycosylation with a donor to acceptor feed ratio of 10:1 showed several peaks corresponding to the molecular masses of tetra-octa-saccharides having one–five Man or GlcN residues with Glc_3_. Further chain-elongation for higher DPs, however, was inhibited by Pi produced from the glycosyl donors, which is a naturally occurring substrate for phosphorolysis by phosphorylase catalysis.

**Figure 11:**
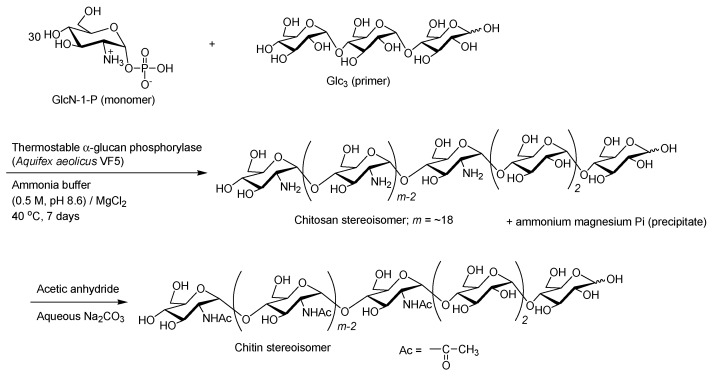
Thermostable phosphorylase-catalyzed enzymatic polymerization of α-d-glucosamine 1-phosphate to produce chitosan stereoisomer and subsequent *N*-acetylation to produce chitin stereoisomer.

An attempt was made to remove this Pi as a precipitate in the thermostable phosphorylase-catalyzed glucosaminylation by conducting the reaction in an ammonium buffer (0.5 M, pH 8.6) containing MgCl_2_, as it has been reported that Pi forms an insoluble salt with ammonium and magnesium ions [74]. Consequently, the thermostable phosphorylase-catalyzed polymerization in the buffer system at 40 °C for 7 days of GlcN-1-P with the Glc_3_ primer (30:1) successfully occurred to produce the α(1→4)-linked glucosamine polymer with a DP of the GlcN units of ~20 (*M*_n_ = 3760 ), which corresponded to the structure of a chitosan stereoisomer, a non-natural aminopolysaccharide (Figure 11) [75]. This product was then converted into a chitin stereoisomer, also a non-natural aminopolysaccharide, by *N*-acetylation using acetic anhydride (Figure 11). The enzymatic copolymerization of Glc-1-P with GlcN-1-P using thermostable phosphorylase catalysis was also performed under the same conditions to obtain a non-natural heteroaminopolysaccharide composed of Glc/GlcN units [76].

## 5. Preparation of Amylose Supramolecular Materials by Phosphorylase-Catalyzed Enzymatic Polymerization

Amylose constructs left-handed helical conformation and forms inclusion complexes with various guest molecules, typically monomeric and oligomeric low molecular weight compounds [77]. Accordingly it is suitable for incorporation into high-performance polymeric materials. The driving force for this binding of guest molecules is hydrophobic interactions, as the inside of the amylose helix is hydrophobic, and therefore, in aqueous solvents, hydrophobic guest molecules will be spontaneously included in the amylose cavity. On the other hand, little has been reported regarding the formation of inclusion complexes between amylose and higher molecular weight polymeric guest molecules [78,79,80,81,82,83,84,85,86,87]. The difficulty in the inclusion of these polymeric guest molecules into the amylose cavity arises from the necessity for a weak hydrophobic interaction to drive the complexation, and amylose does therefore not have sufficient power to direct the inclusion of long polymeric chains into its cavity.

**Figure 12:**
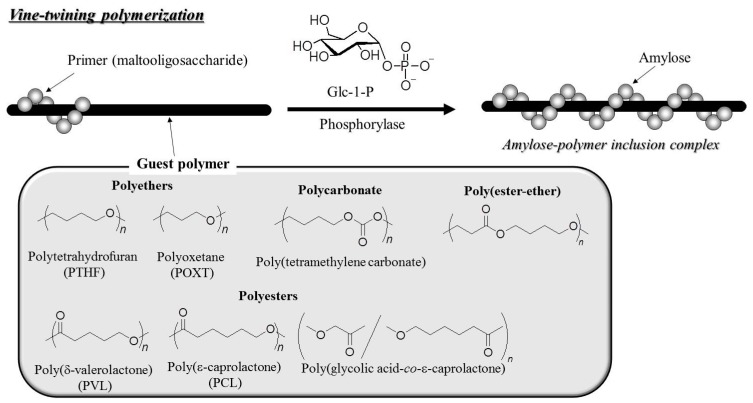
Image of vine-twining polymerization to produce amylose–polymer inclusion complexes and typical guest polymers that have been employed in this system.

It has been reported that phosphorylase-catalyzed enzymatic polymerization to form the shorter α(1→4)-glucan (maltooligosaccharide) to the longer α(1→4)-glucan (amylose) induces inclusion complexation with hydrophobic guest polymers [15,16,34,88,89,90,91,92,93]. Such an efficient method for the formation of amylose-polymer inclusion complexes was achieved by the phosphorylase-catalyzed enzymatic polymerization of amylose in a dispersion of hydrophobic polymers in aqueous buffer solvents. The process of this synthetic method for the generation of such inclusion complexes is similar to the way that vines of plants grow and twine around a rod (Figure 12), which is why this polymerization approach has been named “vine-twining polymerization”. Hydrophobic polyethers, such as poly(tetrahydrofuran) (PTHF), poly(oxetane) (POXT)) [94,95]; polyesters, such as, poly(δ-valerolactone) (PVL), poly(ε-caprolactone) (PCL), poly(glycolic acid-*co*-ε-caprolactone)) [96,97,98]; polycarbonates, such as, poly(tetramethylene carbonate) [99]; and poly(ester-ether)s (–CH_2_CH_2_C(C=O)OCH_2_CH_2_CH_2_CH_2_–) [97] have been observed to act as guest polymers and have been included into amylose using this polymerization technique (Figure 12). Vine-twining polymerization does not produce inclusion complexes with hydrophilic polymers, such as PEG and the other poly(ester–ether)s, owing to insufficient interactions between the polymer and the cavity of the amylose. In addition, the inclusion via vine-twining polymerization has not been achieved with strongly hydrophobic polymers like poly(oxepane), due to their aggregation in aqueous buffer solvents as a result of their longer alkyl chains when compared to PTHF and POXT. It can therefore be concluded that the moderate hydrophobicity of guest polymers is the important factor in determining whether or not amylose will form inclusion complexes during a vine-twining polymerization.

**Figure 13:**
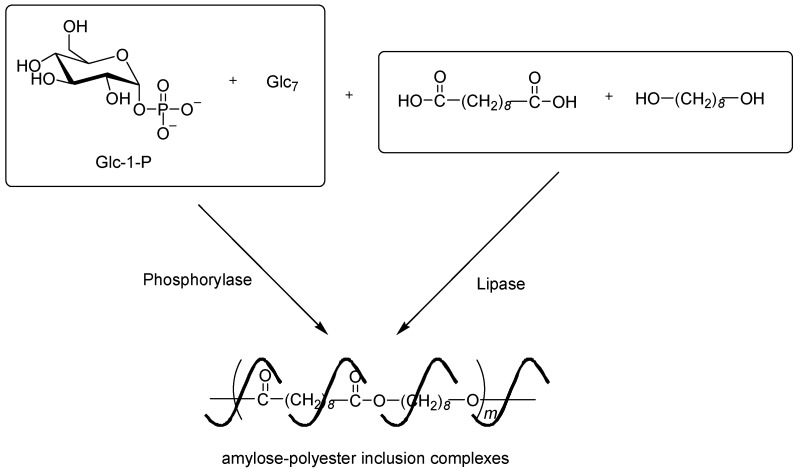
Parallel enzymatic polymerization system to produce inclusion complex from amylose and strongly hydrophobic polyester.

An attempt at a parallel enzymatic polymerization system was made to achieve the formation of an inclusion complex with a strongly hydrophobic polyester [100]. In this system, two enzymatic polymerizations, the phosphorylase-catalyzed enzymatic polymerization of Glc-1-P from the Glc_7_ primer to produce amylose, and the lipase-catalyzed polycondensation of a diol (1,8-octanediol) and a dicarboxylic acid (sebabic acid) to produce a strongly hydrophobic aliphatic polyester as the guest polymer [101,102], were simultaneously conducted in an aqueous buffer solvent (Figure 13). Success was achieved using this approach, and consequently, the analytical results of the reaction fully supported the formation of an inclusion complex between amylose and the polyester.

Amylose showed the selective inclusion behavior in certain structures, molecular weight distributions, and chiralities of the guest polymers. [103,104,105]. For example, poly(l-lactide) (PLLA) with lower *M*_n_, *ca.* 1000–3000 was included in the cavity of amylose in the vine-twining polymerization process, to form the amylose-PLLA inclusion complex, while PLLA with higher *M*_n_, *ca.* >6000, and its stereoisomers, poly(d-lactide) (PDLA) and poly(dl-lactide), were not recognized as guest polymers and were therefore not taken up by the amylose [106].

**Figure 14:**
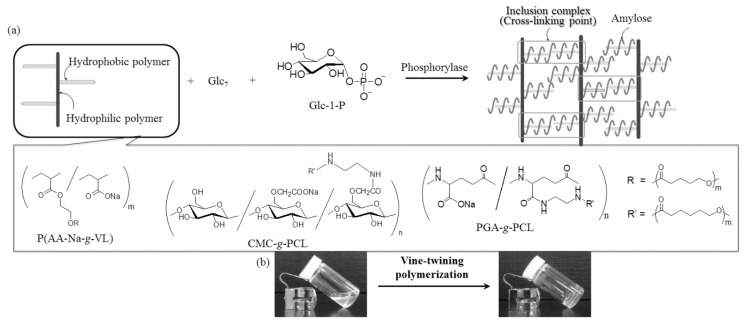
(**a**) Preparation of amylose supramolecular network materials by vine-twining polymerization using graft copolymers having hydrophilic main-chains and hydrophobic guest graft chains and (**b**) photographs before and after vine-twining polymerization.

Amylose supramolecular network hydrogels were fabricated through the formation of vine-twined inclusion complexes of amylose during the phosphorylase-catalyzed polymerization. In these studies, the designed graft copolymers, such as poly(acrylic acid sodium salt-*graft*-δ-valerolactone) (PAA-Na-*g*-PVL), CMC-*graft*-PCL (CMC-*g*-PCL), and poly(γ-glutamic acid-*graft*-ε-caprolactone) (PGA-*g*-PCL) were employed as guest polymers (Figure 14a) [107,108,109], while the hydrophilic main-chains, PAA, CMC, and PGA, acted as the main components of the hydrogels. When the vine-twining polymerization was carried out in the presence of these guest graft copolymers in aqueous acetate buffer solution, the reaction mixtures yielded the supramolecular network hydrogels through inclusion of the graft chains with amylose to form supramolecular copolymers (Figure 14b). The intermolecular inclusion complexes could act as cross-linking points to construct higher-order supramolecular network structures in the hydrogels.

The ability of the hydrogel of PAA-Na-*g*-PVL to reversibly facilitate enzymatic disruption and reproduction was successfully demonstrated by the combined use of β-amylase-catalyzed hydrolysis of the amylose component in the hydrogel, followed by its reformation by subsequent phosphorylase-catalyzed enzymatic polymerization [107]. By the two successive enzymatic reactions, therefore, enzymatically recyclable behavior of the hydrogel at the molecular level was observed. A film was further formed by adding water followed by drying to a powdered sample prepared by lyophilization of the hydrogel from CMC-*g*-PCL [108]. 

The macroscopic interfacial healing of a supramolecular hydrogel consisting of PGA-*g*-PCL was observed through phosphorylase-catalyzed enzymatic polymerization [109]. The hydrogel formed initially from vine-twining polymerization was cut into two pieces, and a sodium acetate buffer solution containing Glc-1-P and phosphorylase was dropped on the surfaces of the hydrogel pieces. After the surfaces were placed in contact with one another, the materials were left standing at 40 °C for 6 h, over which time the enzymatic polymerization proceeded, such that the two hydrogel pieces fused at the point of contact. The healing of the gels on a macroscopic level was induced by the complexation of the enzymatically-produced amyloses with the PCL graft chains at the interface. Porous cryogels were fabricated by the lyophilization of the hydrogel, while ion gels could be prepared by soaking the hydrogels in an ionic liquid of 1-butyl-3-methylimidazolium chloride.

**Figure 15:**
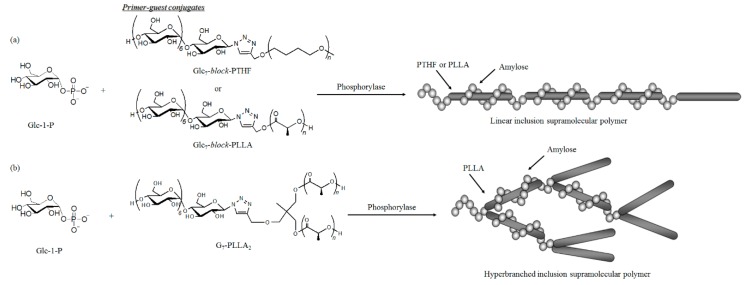
Preparation of (**a**) linear and (**b**) hyperbranched supramolecular polymers by vine-twining polymerization using primer–guest conjugates.

Supramolecular polymers comprised of amylose-PTHF and amylose-PLLA inclusion complexes were successfully fabricated by vine-twining polymerizations using Glc_7_-*block*-PTHF and Glc_7_-*block*-PLLA, respectively, as primer–guest conjugates (Figure 15a) [110,111]. In these systems, a propagating amylose chain initiated by phosphorylase catalysis from a Glc_7_ conjugate could potentially include a guest polymer segment from another conjugate, whereby the successive occurrence of such an event would give rise to linear inclusion supramolecular polymers.

Similarly, vine-twining polymerization using a branched Glc_7_-PLLA_2_ conjugate would produce a hyperbranched inclusion supramolecular polymer composed of amylose-PLLA inclusion complexes (Figure 15b) [112]. This hyperbranched product could then form an ion gel with 1-butyl-3-methylimidazolium chloride, which could be further converted into a hydrogel upon exchange of the dispersion media. The lyophilization of the hydrogel resulted in the fabrication of a cryogel with a porous morphology. The gelation behavior of the hyperbranched supramolecular polymer was a result of its highly extended structure, whereas the aforementioned linear supramolecular polymer did not exhibit such gelation behavior.

The relative chain orientations of amylose and the two stereoisomers of PLA in the inclusion complexes formed in phosphorylase-catalyzed enzymatic polymerizations were investigated by using four different primer–guest conjugates, which consisted of a Glc_7_ moiety functionalized at the carboxylate or hydroxy termini of both PLLA and PDLA [113]. The amylose-PLLA supramolecular polymers formed in the enzymatic polymerization in the presence of both of the two PLLA conjugates, suggesting that, regardless of the chain orientation of PLLA, the amylose cavity took up the guest polymer. Interestingly, the amylose–PDLA diblock copolymers did not form inclusion complexes, because of the effects of chirality on the recognition behavior of amylose, which were irrespective of the PDLA chain orientation. The similar left-handed helical direction of amylose and PLLA imply that an inclusion complex will form, whereas the directions of methyl substituents in PLA toward amylose, which are oppositely changed depending on the relative chain orientation, are not the important factor for the complexation.

## 6. Conclusions

This review presents evidence that phosphorylase-catalyzed enzymatic reactions efficiently provide various polysaccharide materials with precisely controlled structures. Such artificial polysaccharides exhibit specific and unique functions, which are very different to those of typical synthetic polymers. Furthermore, this enzymatic approach is commonly identified as a green and sustainable process because it is generally conducted in aqueous media under mild conditions and proceeds without the production of harmful by-products. Accordingly, the phosphorylase-catalyzed methods described herein have the potential to be applied in the environmentally benign production of useful and functional polysaccharide materials in various fields, such as biomedicine and tissue engineering. The author strongly believes that the further development of these enzymatic methods, including phosphorylase catalysis, will provide unique functional materials in the future.

## References

[1] Schuerch C., Mark H.F., Bilkales N., Overberger C.G. (1986). Polysaccharides. Encyclopedia of Polymer Science and Engineering.

[2] Paulsen H. (1982). Advances in selective chemical syntheses of complex oligosaccharides. Angew. Chem. Int. Ed. Engl..

[3] Schmidt R.R. (1986). New methods for the synthesis of glycosides and oligosaccharides—Are there alternatives to the Koenigs-Knorr method?. Angew. Chem. Int. Ed. Engl..

[4] Toshima K., Tatsuta K. (1993). Recent progress in *O*-glycosylation methods and its application to natural products synthesis. Chem. Rev..

[5] Yalpani M. (1988). Polysaccharides: Syntheses, Modifications, and Structure/Property Relations.

[6] Lenz R.W. (1993). Biodegradable polymers. Adv. Polym. Sci..

[7] Klemm D., Heublein B., Fink H.P., Bohn A. (2005). Cellulose: Fascinating biopolymer and sustainable raw material. Angew. Chem. Int. Ed..

[8] Shoda S., Fraser-Reid B.O., Tatsuta K., Thiem J. (2001). Enzymatic glycosylation. Glycoscience Chemistry and Chemical Biology.

[9] Shoda S., Izumi R., Fujita M. (2003). Green process in glycotechnology. Bull. Chem. Soc. Jpn..

[10] Blixt O., Razi N., Fraser-Reid B.O., Tatsuta K., Thiem J. (2008). Enzymatic glycosylation by transferases. Glycoscience Chemistry and Chemical Biology.

[11] Thiem J., Thiem J., Fraser-Reid B.O., Tatsuta K., Thiem J. (2008). Enzymatic glycosylation by glycohydrolases and glycosynthases. Glycoscience Chemistry and Chemical Biology.

[12] Seibel J., Beine R., Moraru R., Behringer C., Buchholz K. (2006). A new pathway for the synthesis of oligosaccharides by the use of non-Leloir glycosyltransferases. Biocatal. Biotransform..

[13] Seibel J., Jordening H.J., Buchholz K. (2006). Glycosylation with activated sugars using glycosyltransferases and transglycosidases. Biocatal. Biotransform..

[14] Kobayashi S., Makino A. (2009). Enzymatic polymer synthesis: An opportunity for green polymer chemistry. Chem. Rev..

[15] Kadokawa J. (2011). Precision polysaccharide synthesis catalyzed by enzymes. Chem. Rev..

[16] Kadokawa J., Kaneko Y. (2013). Engineering of Polysaccharide Materials—By Phosphorylase-Catalyzed Enzymatic Chain-Elongation.

[17] Kitaoka M., Hayashi K. (2002). Carbohydrate-processing phosphorolytic enzymes. Trends Glycosci. Glycotechnol..

[18] Nakai H., Kitaoka M., Svensson B., Ohtsubo K. (2013). Recent development of phosphorylases possessing large potential for oligosaccharide synthesis. Curr. Opin. Chem. Biol..

[19] Puchart V. (2015). Glycoside phosphorylases: Structure, catalytic properties and biotechnological potential. Biotechnol. Adv..

[20] Yanase M., Takaha T., Kuriki T. (2006). α-Glucan phosphorylase and its use in carbohydrate engineering. J. Sci. Food Agric..

[21] Boeck B., Schinzel R. (1996). Purification and characterisation of an α-glucan phosphorylase from the thermophilic bacterium *Thermus thermophilus*. Eur. J. Biochem..

[22] Takaha T., Yanase M., Takata H., Okada S. (2001). Structure and properties of *Thermus aquaticus* α-glucan phosphorylase expressed in *Escherichichia coli*. J. Appl. Glycosci..

[23] Yanase M., Takata H., Fujii K., Takaha T., Kuriki T. (2005). Cumulative effect of amino acid replacements results in enhanced thermostability of potato type L α-glucan phosphorylase. Appl. Environ. Microbiol..

[24] Ziegast G., Pfannemüller B. (1987). Linear and star-shaped hybrid polymers. 4. Phosphorolytic syntheses with di-functional, oligo-functional and multifunctional primers. Carbohydr. Res..

[25] Fujii K., Takata H., Yanase M., Terada Y., Ohdan K., Takaha T., Okada S., Kuriki T. (2003). Bioengineering and application of novel glucose polymers. Biocatal. Biotransform..

[26] Ohdan K., Fujii K., Yanase M., Takaha T., Kuriki T. (2006). Enzymatic synthesis of amylose. Biocatal. Biotransform..

[27] Kitamura S., Salamone C. (1996). Starch polymers, natural and synthetic. The Polymeric Materials Encyclopedia, Synthesis, Properties and Applications.

[28] Kitamura S., Yunokawa H., Mitsuie S., Kuge T. (1982). Study on polysaccharide by the fluorescence method. 2. Micro-brownian motion and conformational change of amylose in aqueous-solution. Polym. J..

[29] Kaneko Y., Kadokawa J., Ito R., Matsuo Y. (2009). Chemoenzymatic synthesis of amylose-grafted polymers. Handbook of Carbohydrate Polymers: Development, Properties and Applications.

[30] Izawa H., Kadokawa J., Kadoakwa J. (2009). Preparation of functional amylosic materials by phosphorylase-catalyzed polymerization. Interfacial Researches in Fundamental and Material Sciences of Oligo- and Polysaccharides.

[31] Omagari Y., Kadokawa J. (2011). Synthesis of heteropolysaccharides having amylose chains using phosphorylase-catalyzed enzymatic polymerization. Kobunshi Ronbunshu.

[32] Kadoakwa J., Smith P.B., Gross R.A. (2012). Synthesis of amylose-grafted polysaccharide materials by phosphorylase-catalyzed enzymatic polymerization. Biobased Monomers, Polymers, and Materials.

[33] Kadoakwa J., Cheng H.N., Gross R.A., Smith P.B. (2013). Synthesis of new polysaccharide materials by phosphorylase-catalyzed enzymatic α-glycosylations using polymeric glycosyl acceptors. Green Polymer Chemistry: Biocatalysis and Materials II.

[34] Kadokawa J. (2014). Chemoenzymatic synthesis of functional amylosic materials. Pure Appl. Chem..

[35] Kobayashi K., Kamiya S., Enomoto N. (1996). Amylose-carrying styrene macromonomer and its homo- and copolymers: Synthesis via enzyme-catalyzed polymerization and complex formation with iodine. Macromolecules.

[36] Narumi A., Kawasaki K., Kaga H., Satoh T., Sugimoto N., Kakuchi T. (2003). Glycoconjugated polymer 6. Synthesis of poly[styrene-*block*-(styrene-*graft*-amylose)] via potato phosphorylase-catalyzed polymerization. Polym. Bull..

[37] Von Braunmühl V., Jonas G., Stadler R. (1995). Enzymatic grafting of amylose from poly(dimethylsiloxanes). Macromolecules.

[38] Kadokawa J., Nakamura Y., Sasaki Y., Kaneko Y., Nishikawa T. (2008). Chemoenzymatic synthesis of amylose-grafted polyacetylenes. Polym. Bull..

[39] Sasaki Y., Kaneko Y., Kadokawa J. (2009). Chemoenzymatic synthesis of amylose-grafted polyacetylene by polymer reaction manner and its conversion into organogel with DMSO by cross-linking. Polym. Bull..

[40] Kaneko Y., Matsuda S., Kadokawa J. (2010). Chemoenzymatic synthesis of amylose-grafted poly(vinyl alcohol). Polym. Chem..

[41] Kamiya S., Kobayashi K. (1998). Synthesis and helix formation of saccharide–poly(l-glutamic acid) conjugates. Macromol. Chem. Phys..

[42] Mazzocchetti L., Tsoufis T., Rudolf P., Loos K. (2014). Enzymatic synthesis of amylose brushes revisited: Details from X-ray photoelectron spectroscopy and spectroscopic ellipsometry. Macromol. Biosci..

[43] Kadokawa J., Gordon N.S. (2011). Facile synthesis of unnatural oligosaccharides by phosphorylase-catalyzed enzymatic glycosylations using new glycosyl donors. Oligosaccharides: Sources, Properties and Applications.

[44] Kadokawa J. (2013). Synthesis of non-natural oligosaccharides by α-glucan phosphorylase-catalyzed enzymatic glycosylations using analogue substrates of α-d-glucose 1-phosphate. Trends Glycosci. Glycotechnol..

[45] Percival M.D., Withers S.G. (1988). Applications of enzymes in the synthesis and hydrolytic study of 2-deoxy-α-d-glucopyranosyl phosphate. Can. J. Chem..

[46] Evers B., Mischnick P., Thiem J. (1994). Synthesis of 2-deoxy-α-d-arabino-hexopyranosyl phosphate and 2-deoxy-maltooligosaccharides with phosphorylase. Carbohydr. Res..

[47] Evers B., Thiem J. (1997). Further syntheses employing phosphorylase. Bioorg. Med. Chem..

[48] Nawaji M., Izawa H., Kaneko Y., Kadokawa J. (2008). Enzymatic synthesis of α-d-xylosylated maltooligosaccharides by phosphorylase-catalyzed xylosylation. J. Carbohydr. Chem..

[49] Nawaji M., Izawa H., Kaneko Y., Kadokawa J. (2008). Enzymatic α-glucosaminylation of maltooligosaccharides catalyzed by phosphorylase. Carbohydr. Res..

[50] Kawazoe S., Izawa H., Nawaji M., Kaneko Y., Kadokawa J. (2010). Phosphorylase-catalyzed *N*-formyl-α-glucosaminylation of maltooligosaccharides. Carbohydr. Res..

[51] Stephen A.M., Phillips G.O., Williams P.A. (2006). Food Polysaccharides and Their Applications.

[52] Matsuda S., Kaneko Y., Kadokawa J. (2007). Chemoenzymatic synthesis of amylose-grafted chitosan. Macromol. Rapid Comm..

[53] Kaneko Y., Matsuda S., Kadokawa J. (2007). Chemoenzymatic syntheses of amylose-grafted chitin and chitosan. Biomacromolecules.

[54] Omagari Y., Matsuda S., Kaneko Y., Kadokawa J. (2009). Chemoenzymatic synthesis of amylose-grafted cellulose. Macromol. Biosci..

[55] Omagari Y., Kaneko Y., Kadokawa J. (2010). Chemoenzymatic synthesis of amylose-grafted alginate and its formation of enzymatic disintegratable beads. Carbohydr. Polym..

[56] Arimura T., Omagari Y., Yamamoto K., Kadokawa J. (2011). Chemoenzymatic synthesis and hydrogelation of amylose-grafted xanthan gums. Int. J. Biol. Macromol..

[57] Kadokawa J., Arimura T., Takemoto Y., Yamamoto K. (2012). Self-assembly of amylose-grafted carboxymethyl cellulose. Carbohydr. Polym..

[58] Hatanaka D., Takemoto Y., Yamamoto K., Kadokawa J. (2013). Hierarchically self-assembled nanofiber films from amylose-grafted carboxymethyl cellulose. Fibers.

[59] Calder P.C. (1991). Glycogen structure and biogenesis. Int. J. Biochem..

[60] Manners D.J. (1991). Recent developments in our understanding of glycogen structure. Carbohydr. Polym..

[61] Izawa H., Nawaji M., Kaneko Y., Kadokawa J. (2009). Preparation of glycogen-based polysaccharide materials by phosphorylase-catalyzed chain elongation of glycogen. Macromol. Biosci..

[62] Hinrichs W., Buttner G., Steifa M., Betzel C., Zabel V., Pfannemuller B., Saenger W. (1987). An amylose antiparallel double helix at atomic resolution. Science.

[63] Eisenhaber F., Schulz W. (1992). Monte-carlo simulation of the hydration shell of double-helical amylose—A left-handed antiparallel double helix fits best into liquid water-structure. Biopolymers.

[64] Morimoto N., Ogino N., Narita T., Kitamura S., Akiyoshi K. (2007). Enzyme-responsive molecular assembly system with amylose-primer surfactants. J. Am. Chem. Soc..

[65] Morimoto N., Ogino N., Narita T., Akiyoshi K. (2009). Enzyme-responsive artificial chaperone system with amphiphilic amylose primer. J. Biotechnol..

[66] Morimoto N., Yamazaki M., Tamada J., Akiyoshi K. (2013). Polysaccharide-hair cationic polypeptide nanogels: Self-assembly and enzymatic polymerization of amylose primer-modified cholesteryl poly(l-lysine). Langmuir.

[67] Nishimura T., Mukai S., Sawada S., Akiyoshi K. (2015). Glyco star polymers as helical multivalent host and biofunctional nano-platform. Acs Macro Lett..

[68] Bhuiyan S.H., Rus’d A.A., Kitaoka M., Hayashi K. (2003). Characterization of a hyperthermostable glycogen phosphorylase from *Aquifex aeolicus* expressed in *Escherichia coli*. J. Mol. Catal. B Enzym..

[69] Umegatani Y., Izawa H., Nawaji M., Yamamoto K., Kubo A., Yanase M., Takaha T., Kadokawa J. (2012). Enzymatic α-glucuronylation of maltooligosaccharides using α-glucuronic acid 1-phosphate as glycosyl donor catalyzed by a thermostable phosphorylase from *Aquifex aeolicus* vf5. Carbohydr. Res..

[70] Takata Y., Shimohigoshi R., Yamamoto K., Kadokawa J. (2014). Enzymatic synthesis of dendritic amphoteric α-glucans by thermostable phosphorylase catalysis. Macromol. Biosci..

[71] Takata Y., Yamamoto K., Kadokawa J. (2015). Preparation of pH-responsive amphoteric glycogen hydrogels by α-glucan phosphorylase-catalyzed successive enzymatic reactions. Macromol. Chem. Phys..

[72] Takemoto Y., Izawa H., Umegatani Y., Yamamoto K., Kubo A., Yanase M., Takaha T., Kadokawa J. (2013). Synthesis of highly branched anionic α-glucans by thermostable phosphorylase-catalyzed α-glucuronylation. Carbohydr. Res..

[73] Shimohigoshi R., Takemoto Y., Yamamoto K., Kadokawa J. (2013). Thermostable α-glucan phosphorylase-catalyzed successive α-mannosylations. Chem. Lett..

[74] Borgerding J. (1972). Phosphate deposits in digestion systems. J. Water Pollut. Control Fed..

[75] Kadokawa J., Shimohigoshi R., Yamashita K., Yamamoto K. (2015). Synthesis of chitin and chitosan stereoisomers by thermostable α-glucan phosphorylase-catalyzed enzymatic polymerization of α-d-glucosamine 1-phosphate. Org. Bimol. Chem..

[76] Yamashita K., Yamamoto K., Kadoakwa J. (2015). Synthesis of non-natural heteroaminopolysaccharides by α-glucan phosphorylase-catalyzed enzymatic copolymerization: α(1–>4)-linked glucosaminoglucans. Biomacromolecules.

[77] Sarko A., Zugenmaier P., French A.D., Gardner K.H. (1980). Crystal structures of amylose and its derivatives. Fiber Diffraction Methods.

[78] Shogren R.L., Greene R.V., Wu Y.V. (1991). Complexes of starch polysaccharides and poly(ethylene-*co*-acrylic acid): Structure and stability in solution. J. Appl. Polym. Sci..

[79] Shogren R.L. (1993). Complexes of starch with telechelic poly(ε-caprolactone) phosphate. Carbohydr. Polym..

[80] Star A., Steuerman D.W., Heath J.R., Stoddart J.F. (2002). Starched carbon nanotubes. Angew. Chem. Int. Ed..

[81] Ikeda M., Furusho Y., Okoshi K., Tanahara S., Maeda K., Nishino S., Mori T., Yashima E. (2006). A luminescent poly(phenylenevinylene)-amylose composite with supramolecular liquid crystallinity. Angew. Chem. Int. Ed..

[82] Kida T., Minabe T., Okabe S., Akashi M. (2007). Partially-methylated amyloses as effective hosts for inclusion complex formation with polymeric guests. Chem. Commun..

[83] Kaneko Y., Kyutoku T., Shimomura N., Kadokawa J. (2011). Formation of amylose-poly(tetrahydrofuran) inclusion complexes in ionic liquid media. Chem. Lett..

[84] Rachmawati R., Woortman A.J.J., Loos K. (2013). Facile preparation method for inclusion complexes between amylose and polytetrahydrofurans. Biomacromolecules.

[85] Kumar K., Woortman A.J.J., Loos K. (2013). Synthesis of amylose-polystyrene inclusion complexes by a facile preparation route. Biomacromolecules.

[86] Rachmawati R., Woortman A.J.J., Loos K. (2013). Tunable properties of inclusion complexes between amylose and polytetrahydrofuran. Macromol. Biosci..

[87] Rachmawati R., Woortman A.J.J., Loos K. (2014). Solvent-responsive behavior of inclusion complexes between amylose and polytetrahydrofuran. Macromol. Biosci..

[88] Kaneko Y., Kadokawa J. (2005). Vine-twining polymerization: A new preparation method for well-defined supramolecules composed of amylose and synthetic polymers. Chem. Rec..

[89] Kaneko Y., Kadokawa J. (2006). Synthesis of nanostructured bio-related materials by hybridization of synthetic polymers with polysaccharides or saccharide residues. J. Biomater. Sci., Polym. Ed..

[90] Kaneko Y., Kadokawa J., Lee J.N. (2009). Preparation of polymers with well-defined nanostructure in the polymerization field. Modern Trends in Macromolecular Chemistry.

[91] Kaneko Y., Kadokawa J. (2010). Preparation method for polysaccharide supramolecules using amylose-forming polymerization field: Vine-twining polymerization. Kobunshi Ronbunshu.

[92] Kadokawa J. (2012). Preparation and applications of amylose supramolecules by means of phosphorylase-catalyzed enzymatic polymerization. Polymers.

[93] Kadokawa J. (2013). Architecture of amylose supramolecules in form of inclusion complexes by phosphorylase-catalyzed enzymatic polymerization. Biomolecules.

[94] Kadokawa J., Kaneko Y., Tagaya H., Chiba K. (2001). Synthesis of an amylose-polymer inclusion complex by enzymatic polymerization of glucose 1-phosphate catalyzed by phosphorylase enzyme in the presence of polythf: A new method for synthesis of polymer-polymer inclusion complexes. Chem. Commun..

[95] Kadokawa J., Kaneko Y., Nagase S., Takahashi T., Tagaya H. (2002). Vine-twining polymerization: Amylose twines around polyethers to form amylose-polyether inclusion complexes. Chem. Eur. J..

[96] Kadokawa J., Kaneko Y., Nakaya A., Tagaya H. (2001). Formation of an amylose-polyester inclusion complex by means of phosphorylase-catalyzed enzymatic polymerization of α-d-glucose 1-phosphate monomer in the presence of poly(ε-caprolactone). Macromolecules.

[97] Kadokawa J., Nakaya A., Kaneko Y., Tagaya H. (2003). Preparation of inclusion complexes between amylose and ester-containing polymers by means of vine-twining polymerization. Macromol. Chem. Phys..

[98] Nomura S., Kyutoku T., Shimomura N., Kaneko Y., Kadokawa J. (2011). Preparation of inclusion complexes composed of amylose and biodegradable poly(glycolic acid-*co*-ε-caprolactone) by vine-twining polymerization and their lipase-catalyzed hydrolysis behavior. Polym. J..

[99] Kaneko Y., Beppu K., Kadokawa J. (2008). Preparation of amylose/polycarbonate inclusion complexes by means of vine-twining polymerization. Macromol. Chem. Phys..

[100] Kaneko Y., Saito Y., Nakaya A., Kadokawa J., Tagaya H. (2008). Preparation of inclusion complexes composed of amylose and strongly hydrophobic polyesters in parallel enzymatic polymerization system. Macromolecules.

[101] Kobayashi S., Uyama H., Suda S., Namekawa S. (1997). Dehydration polymerization in aqueous medium catalyzed by lipase. Chem. Lett..

[102] Suda S., Uyama H., Kobayashi S. (1999). Dehydration polycondensation in water for synthesis of polyesters by lipase catalyst. Proc. Jpn. Acad. B, Phys. Biol. Sci..

[103] Kaneko Y., Beppu K., Kadokawa J. (2007). Amylose selectively includes one from a mixture of two resemblant polyethers in vine-twining polymerization. Biomacromolecules.

[104] Kaneko Y., Beppu K., Kyutoku T., Kadokawa J. (2009). Selectivity and priority on inclusion of amylose toward guest polyethers and polyesters in vine-twining polymerization. Polym. J..

[105] Kaneko Y., Beppu K., Kadokawa J. (2009). Amylose selectively includes a specific range of molecular weights in poly(tetrahydrofuran)s in vine-twining polymerization. Polym. J..

[106] Kaneko Y., Ueno K., Yui T., Nakahara K., Kadokawa J. (2011). Amylose’s recognition of chirality in polylactides on formation of inclusion complexes in vine-twining polymerization. Macromol. Biosci..

[107] Kaneko Y., Fujisaki K., Kyutoku T., Furukawa H., Kadokawa J. (2010). Preparation of enzymatically recyclable hydrogels through the formation of inclusion complexes of amylose in a vine-twining polymerization. Chem. Asian J..

[108] Kadokawa J., Nomura S., Hatanaka D., Yamamoto K. (2013). Preparation of polysaccharide supramolecular films by vine-twining polymerization approach. Carbohydr. Polym..

[109] Kadokawa J., Tanaka K., Hatanaka D., Yamamoto K. (2015). Preparation of multiformable supramolecular gels through helical complexation by amylose in vine-twining polymerization. Polym. Chem..

[110] Tanaka T., Sasayama S., Nomura S., Yamamoto K., Kimura Y., Kadokawa J. (2013). An amylose-poly(l-lactide) inclusion supramolecular polymer: Enzymatic synthesis by means of vine-twining polymerization using a primer–guest conjugate. Macromol. Chem. Phys..

[111] Tanaka T., Tsutsui A., Gotanda R., Sasayama S., Yamamoto K., Kadokawa J. (2015). Synthesis of amylose-polyether inclusion supramolecular polymers by vine-twining polymerization using maltoheptaose-functionalized poly(tetrahydrofuran) as a primer–guest conjugate. J. Appl. Glycosci..

[112] Tanaka T., Gotanda R., Tsutsui A., Sasayama S., Yamamoto K., Kimura Y., Kadokawa J. (2015). Synthesis and gel formation of hyperbranched supramolecular polymer by vine-twining polymerization using branched primer-guest conjugate. Polymer.

[113] Tanaka T., Sasayama S., Yamamoto K., Kimura Y., Kadokawa J. (2015). Evaluating relative chain orientation of amylose and poly(l-lactide) in inclusion complexes formed by vine-twining polymerization using primer–guest conjugates. Macromol. Chem. Phys..

